# General Theory for Integrated Analysis of Growth, Gene, and Protein Expression in Biofilms

**DOI:** 10.1371/journal.pone.0083626

**Published:** 2013-12-23

**Authors:** Tianyu Zhang, Breana Pabst, Isaac Klapper, Philip S. Stewart

**Affiliations:** 1 Department of Mathematical Sciences, Montana State University, Bozeman, Montana, United States of America; 2 Department of Chemical and Biological Engineering, Montana State University, Bozeman, Montana, United States of America; 3 Department of Mathematics, Temple University, Philadelphia, Pennsylvania, United States of America; 4 Center for Biofilm Engineering, Montana State University, Bozeman, Montana, United States of America; Ghent University, Belgium

## Abstract

A theory for analysis and prediction of spatial and temporal patterns of gene and protein expression within microbial biofilms is derived. The theory integrates phenomena of solute reaction and diffusion, microbial growth, mRNA or protein synthesis, biomass advection, and gene transcript or protein turnover. Case studies illustrate the capacity of the theory to simulate heterogeneous spatial patterns and predict microbial activities in biofilms that are qualitatively different from those of planktonic cells. Specific scenarios analyzed include an inducible GFP or fluorescent protein reporter, a denitrification gene repressed by oxygen, an acid stress response gene, and a quorum sensing circuit. It is shown that the patterns of activity revealed by inducible stable fluorescent proteins or reporter unstable proteins overestimate the region of activity. This is due to advective spreading and finite protein turnover rates. In the cases of a gene induced by either limitation for a metabolic substrate or accumulation of a metabolic product, maximal expression is predicted in an internal stratum of the biofilm. A quorum sensing system that includes an oxygen-responsive negative regulator exhibits behavior that is distinct from any stage of a batch planktonic culture. Though here the analyses have been limited to simultaneous interactions of up to two substrates and two genes, the framework applies to arbitrarily large networks of genes and metabolites. Extension of reaction-diffusion modeling in biofilms to the analysis of individual genes and gene networks is an important advance that dovetails with the growing toolkit of molecular and genetic experimental techniques.

## Introduction

Reporter gene [1]–[5], transcriptomic [6]–[11], and proteomic [12]–[16] technologies have made it possible to measure gene and protein expression in microbial biofilms. How can differences in biofilm gene expression, both in comparison to planktonic cells and in space and time within the biofilm, be understood? Here we provide a general theoretical framework for addressing this question. At the core of the model are reaction-diffusion equations that account for microscale concentration gradients within the biofilm. It is these differences in local concentrations that underpin differences in local growth, gene, and protein expression. Biofilm models incorporating reaction-diffusion analyses date back to the mid-1970s [17]–[19]). These models have been used to simulate and understand such phenomena as overall substrate fluxes in wastewater treatment processes [20], [21], species competition and coexistence [21], [22], the heterogeneous architecture of biofilms [23], [24], antimicrobial penetration and efficacy [25], [26], microbially influenced corrosion [27], [28], pH gradients in dental plaque [29], and mineral precipitation [30]. Apart from certain models of quorum sensing in biofilms [31], [32], there have not been attempts to model the dynamic spatiotemporal expression of specific genes or proteins in microbial biofilms. The overall goal of this work was to construct the first general computational model of spatiotemporal gene (mRNA) and protein expression in microbial biofilms. This aim was achieved and is illustrated with a few case studies.

## Materials and Methods

### Equations in general form

Consider a single-species microbial biofilm. The unknown variables are the concentrations of 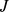 metabolites denoted by a vector 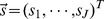, 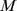 gene transcripts (mRNA) denoted by a vector 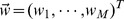, 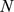 proteins denoted by a vector 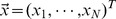, and the velocity due to the growth of the biofilm 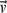. The spatial variable 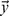 is in a time-varying domain 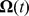 whose boundary is moving in the normal direction with velocity 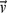. 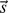 is assumed at equilibrium so it does not depend on time explicitly and is a function of 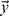 only, and 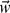 and 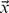 are functions of time 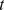 and 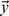. The governing equations written in general coordinate are 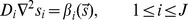
(1)

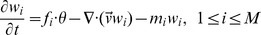
(2)

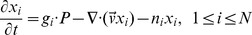
(3)

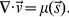
(4)


Here 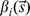 is the reaction (consumption or production) rate of the 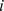-th metabolite, 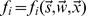 is the fraction of total mRNA synthesis devoted to 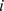-th gene, 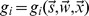 is the fraction of total protein synthesis devoted to 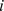-th protein, 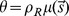 and 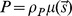 are the overall rates of synthesis of mRNA and protein respectively, 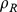 and 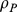 are the mRNA and protein densities, and 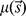 is the specific growth rate of the organism. This formulation assumes that all protein or mRNA synthesis is growth-associated, a restriction that could easily be relaxed to allow for non-growth-associated anabolism. 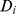 is the effective diffusion coefficient of the 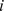-th metabolite in the biofilm, and 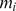 and 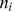 are the turnover rates of the 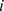-th gene transcript and 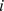-th protein, respectively. 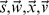 also satisfy appropriate boundary conditions which are problem dependent.

Eqn. (1) is a statement of the balance of reaction and diffusion for each metabolite. This balance determines the spatial variation in the concentration of these substrates or products. Eqns. (2) and (3) are differential material balances on gene transcripts and proteins, respectively. The four terms, from left to right, are accumulation, synthesis, advection, and turnover. The advection term arises from the fact that as cells grow, they push and displace neighboring cells. When a cell is displaced its mRNAs and proteins move with it and so to determine spatial patterns it is necessary to account for this motion. Eqn. (4) is the balance on overall biomass that relates the local change in the advective velocity, 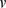, to the local growth rate within the biofilm [22].

We consider either first order or zero order kinetics for metabolite reaction and biofilm growth. In the case of only one metabolite, the corresponding expressions are 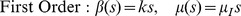


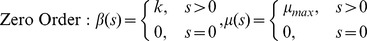



where 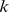 is the reaction rate coefficient, 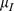 is a growth rate coefficient, and 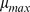 is the maximum specific growth rate. These two kinetic models provide convenient mathematical bounds on the expected saturation behavior of Monod or Michaelis-Menten kinetic forms. We have implemented both zero and first order kinetic models in the case studies partly to underscore the flexible nature of this theoretical framework. Below we will consider four different cases all in one spatial dimension. In three cases, the biofilm is modeled as a flat stab with spatial variable 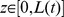, where 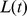 is the biofilm thickness. In one case the biofilm is modeled as a hemispherical cluster of radius 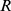. All of the boundary conditions are conventional requirements for either i) no flux or zero velocity at a point of symmetry or impermeability, or ii) imposed bulk fluid concentration. The nomenclature is given in **Table S1**.

### Inducible GFP

This case study is motivated by experimental work with *Pseudomonas aeruginosa* colony biofilms [33]–[35]. In this system, the bacteria are inoculated onto a polymer membrane resting on a tryptic soy agar plate. The bacteria multiply. After 24 h, the membrane is transferred to a fresh plate. After 48 h, bacterial cells are densely aggregated in a hydrated matrix with a thickness of approximately 100 to 200 microns. These 2-day-old colony biofilms have been shown to be highly tolerant to antibiotics [35] and to contain oxygen concentration gradients [34], [35] and in these ways resemble biofilms developed in other in vitro systems.

To investigate spatial patterns of growth and gene expression in colony biofilms, bacterial strains containing inducible green fluorescent proteins have been employed. Typically, the biofilm is grown in the absence of the inducer (arabinose) for 48 h, then transferred to a plate containing inducer. Cells that are capable of de novo protein synthesis begin to synthesize the fluorescent protein. The spatial pattern of GFP within the biofilm can be visualized and quantified by embedding, sectioning, and imaging with fluorescence microscopy. To investigate fluorescent protein decay, the biofilm is grown under inducing conditions for 48 h, then transferred to non-inducing conditions.

Oxygen is the limiting substrate for this aerobic microorganism (the medium contains no nitrate or arginine). We therefore choose one metabolite, oxygen (denoted by 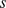), and one protein, GFP (denoted by 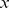), thus 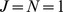. Zero order kinetics are used. The governing equations for a slab biofilm with uniform thickness are given by 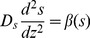
(5)

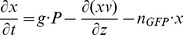
(6)

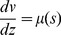
(7)

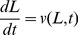
(8)


**Figure 1:**
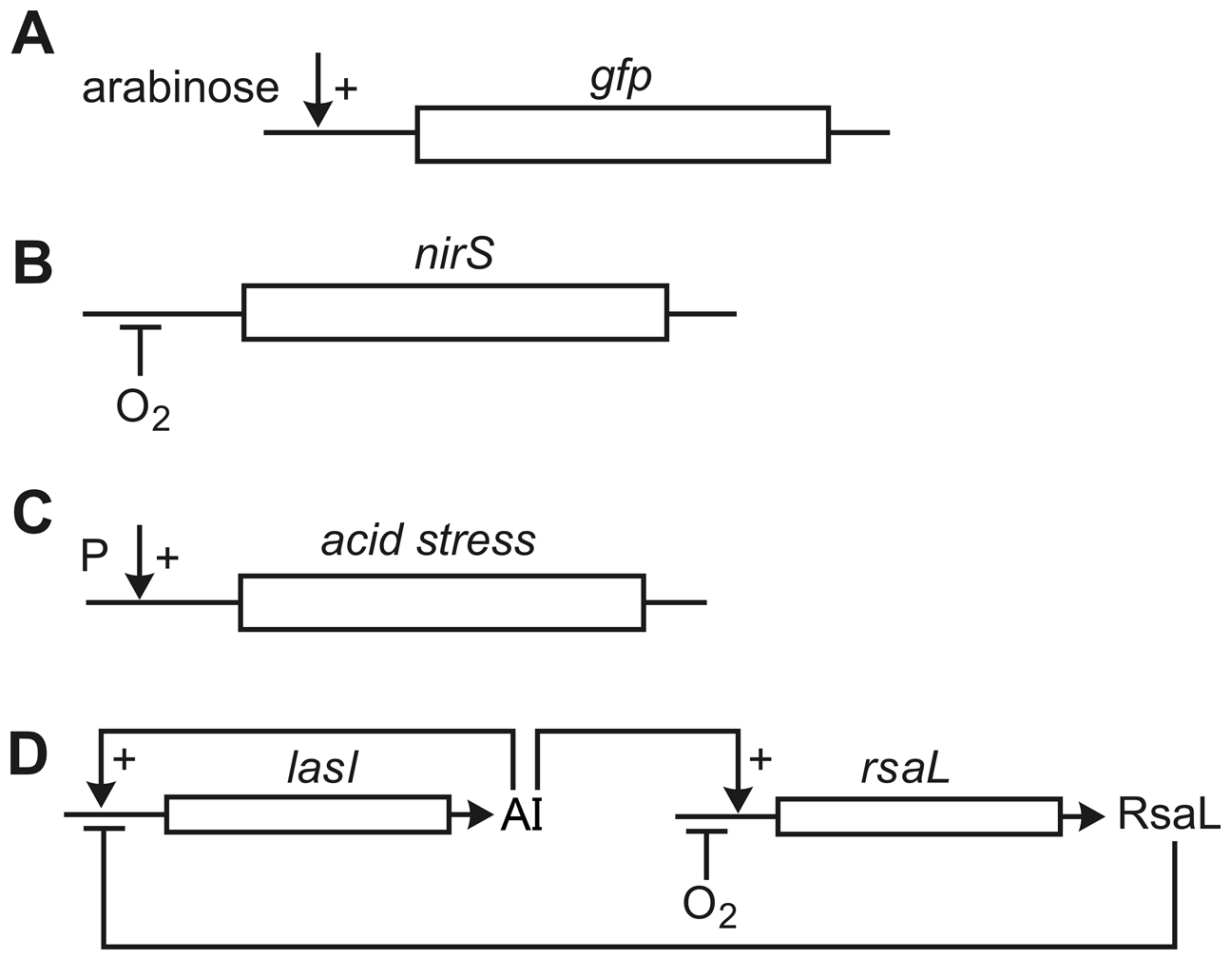
Diagram of simulated genetic circuits. A, inducible GFP. A stable GFP is under the control of the arabinose-inducible 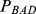 promoter; B, acid stress response. An acidic metabolic product, P, positively regulates the expression of an acid stress gene; C, nitrite reductase induction by low oxygen. Oxygen represses transcription of *nirS*; D, quorum sensing circuit. A homoserine lactone synthase, *lasI*, produces a quorum sensing signal molecule or autoinducier, AI. AI positively regulates the production of its own biosynthetic enzyme. This simple feedback loop gives the essential autoinduction property. AI also positively regulates the expression of a second gene, *rsaL*, which is itself a negative regulator of many of the quorum sensing controlled genes. Like the *nirS* circuit, *rsaL* is expressed under conditions of low oxygen and repressed under conditions of high oxygen.

Here 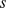 and velocity 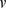 satisfy the following boundary conditions 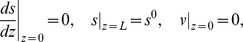
and 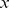 and the biofilm thickness 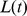 satisfy the following initial conditions >
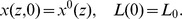




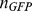
 is the turnover rate of GFP. The fraction factor 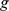 describing the expression of GFP (Figure 1A) is defined by 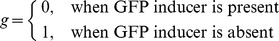



### Denitrification

Denitrification refers to the use of oxidized nitrogen species such as nitrate or nitrite as alternative electron acceptors for microbial respiration. Denitrification is energetically less favorable than respiration of oxygen, so denitrification genes are typically repressed in the presence of oxygen (Figure 1B). This case study was inspired by the experimental work of Kofoed et al who created an artificial biofilm by immobilizing a denitrifying bacterium, *Pseudomonas stutzeri*, in agarose gel slabs [36]. They experimentally determined the spatial pattern of expression of a gene involved in denitrification, *nirS*, which encodes a nitrite reductase. In this case we have two metabolites, oxygen (denoted by 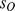) and nitrite (denoted by 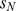), and one gene, *nirS* (denoted by 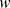), therefore 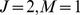. First order kinetics are used and it is assumed that the biofilm has a constant thickness 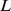. The justification for assuming constant thickness is that the artificial biofilm gels do not expand in time. The governing equations are given by 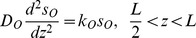
(9)

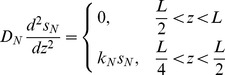
(10)

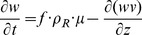
(11)


The boundary conditions are 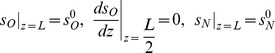


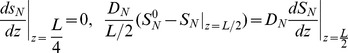



The intervals of applicability for Eqns. (9) and (10) and location of boundaries reflected in the boundary conditions correspond to the following interpretation of the experimental results shown in Figure 1 of [36]. The zone of oxygen respiration was estimated to occupy the top 200 microns of the artificial biofilm. Since the overall biofilm thickness was 400 microns, this corresponds to that half of the biofilm adjacent to the oxygen source. The zone of active denitrification was estimated to occupy one quarter of the biofilm just below the oxic zone. The fraction of total mRNA synthesis devoted to *nirS* decays with increasing oxygen concentration according to 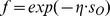



Here 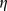 is a factor characterizing the suppressing effect of oxygen on *nirS*. The function 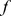 describes an exponential repression of *nirS* gene expression by oxygen (see Figure S1). At relatively high concentrations of oxygen (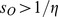), there will be little expression of this gene. As the concentration of oxygen becomes small (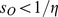), the *nirS* gene will be expressed. The growth rate 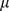 is defined by 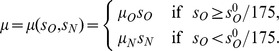
where 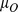 and 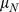 are the specific growth rate coefficients corresponding to oxygen and nitrite, respectively. The reaction coefficient 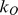 is chosen such that the oxygen concentration drops from its bulk value of 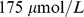 to 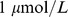 at 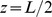. The growth rate function, 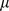, stipulates that growth depends on oxygen concentration when oxygen is present (concentrations greater than or equal to 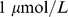 or 1/175 of the bulk fluid concentration) and depends on nitrite concentration when oxygen is absent (concentrations less than 1/175 of the bulk fluid concentration). Transcript turnover is neglected.

### Acid Stress Response

Global transcriptional profiling has provided evidence for acid stress in staphylococcal biofilms fermenting glucose [6], [11]. It is this observation that motivates this case study. In this case we have two metabolites, glucose (denoted by 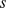) and lactate (denoted by 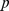), and one acid induced gene (denoted by 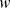), therefore 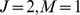. Lactate is an acidic waste product that will be assumed to induce the expression of the gene (Figure 1C). First order kinetics are used and a detachment term is included to moderate biofilm thickness. We also assume a spherical symmetry of the problem with the only spatial variation in the radial direction. The radius of the biofilm cluster is denoted by 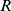. Spherical coordinates were chosen to make the point that the theory is not geometry dependent and because biofilm clusters do often have rounded or hemispherical shapes.The governing equations are given by 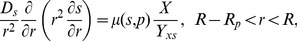
(12)

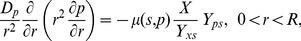
(13)

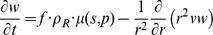


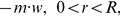
(14)

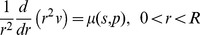
(15)

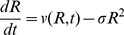
(16)


The last term in Eqn. (16) is the detachment term which uses a conventional squared dependence on biofilm dimension [37]. The boundary conditions are 
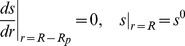


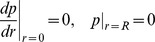



Here 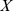 is the cell density in the biofilm, 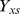 is the yield coefficient of cell on glucose, 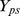 is the yield coefficient of lactate on glucose, 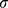 is the detachment coefficient, and 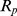 is the specified penetration depth of glucose. The fraction factor 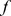 is defined by 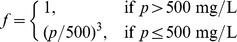



The growth rate 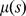 is defined by 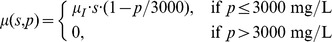



The form of 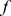 was assumed to allow for full expression of the gene above a certain product concentration (500 mg/L in this case) with only weak expression at very low product concentrations (Figure S1). The growth rate function incorporates product inhibition. At high product concentrations, the bacteria slow down and cease growth.

### Quorum Sensing

We consider a simplified quorum sensing circuit based on the homoserine lactone signaling of *P. aeruginosa*. The product of the signal synthase, *lasI*, synthesizes an autoinducer that stimulates it own expression [38]. A second gene, *rsaL*, while also under quorum sensing control negatively regulates the expression of *lasI* [39], [40]. The expression of *rsaL* is also repressed by oxygen [41], [42]. Thus this circuit, diagrammed in Figure 1D, involves two substrates and two genes which are interlinked through two positive and two negative interactions. The expected outcomes from such a circuit operating in a biofilm are not intuitive. In this case we have two metabolites, oxygen (denoted by 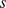) and an autoinducer molecule (denoted by 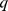), and two genes, *lasI* (denoted by 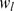) and *rsaL* (denoted by 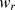), therefore 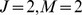. Zero order kinetics are used. The governing equations are given by 
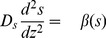
(17)

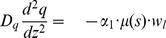
(18)

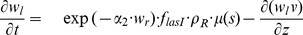
(19)

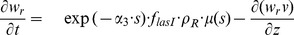
(20)

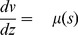
(21)

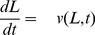
(22)


The exponential terms in Eqns. (19) and (20) provide strong repression of *lasI* expression by *rsaL* and strong repression of *rsaL* expression by oxygen, respectively. The boundary conditions are 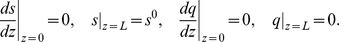



Here 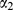 and 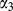 are coefficients characterizing the suppressing effect of *rsaL* on *lasI* and oxygen on *rsaL*, respectively. The fraction factor 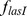 is defined by 
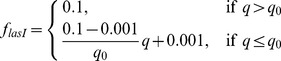



This functional form for 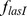, which is graphed in **Figure S1**, provides the positive feedback of increasing expression of *lasI* with increasing autoinducer concentration. Note that there is some small basal expression of *lasI* even when 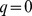. This allows for autoinduction even when there is initially no *lasI* expressed.

Below are the equations for the quorum sensing case in batch culture. These equations simulate planktonic growth and are analyzed to allow for a comparison of behaviors between planktonic and biofilm growth modes. A key difference between these scenarios is that the planktonic culture is continuously aerated, as described by Eqn. (23), and so the planktonic culture is not expected to experience the same degree of oxygen limitation as does the biofilm. The functional dependencies for gene expression are the same for the planktonic and biofilm simulations. 
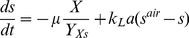
(23)

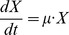
(24)

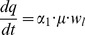
(25)

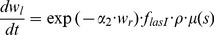
(26)

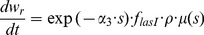
(27)


Here 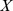 is the cell density in the batch culture, 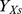 is the yield coefficient, 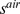 is the oxygen concentration in the air, 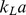 is the mass transfer coefficient for oxygen exchange between the liquid and air.

### Numerical method

All equations are nondimensionalized with characteristic length scale 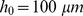 and time scale 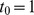 hour. For computational convenience, a new spatial variable 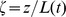 is introduced to change the moving boundary problem with 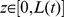 to a fixed domain problem with 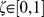 (**Text S1**). Among the governing equations (5)–(22), (5), (9), (10), and (17) are solved analytically, the velocity 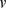 is obtained by numerically integrating the growth term 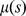 with trapezoidal rule, the biofilm thickness 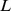 is obtained by integrating the velocity 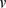 with forward Euler method, and the rest of the equations are solved by a finite difference method with uniform spatial grid size 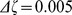 and time step size 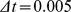. The backward Euler method is used for the time discretization and an upwind scheme is used for the advection term. The ordinary differential equations (23)–(27) for the quorum sensing case in batch culture are solved by MATLAB function ode45. Parameter values used in the simulations can be found in **Tables S2–S5**.

### Image analysis

Image analysis was performed on micrographs of colony biofilm sections containing GFP collected in previous experimental studies [33], [34]. Using the linescan function in Metamorph Version 7.7.0.0, intensity versus distance curves were constructed for three cross-sections of each micrograph. Numerical integration using left-hand Riemann sums was performed to approximate the area under the curves.

## Results

We have derived a general theory for predicting the spatiotemporal evolution of gene transcript and protein expression within bacterial biofilms. Here the utility and flexibility of this theoretical framework is illustrated through comparisons to experimental systems encompassing a range of biological phenomena including stratified growth, protein turnover, repression of gene expression by oxygen, induction of gene expression by acid stress, and quorum sensing.

**Figure 2:**
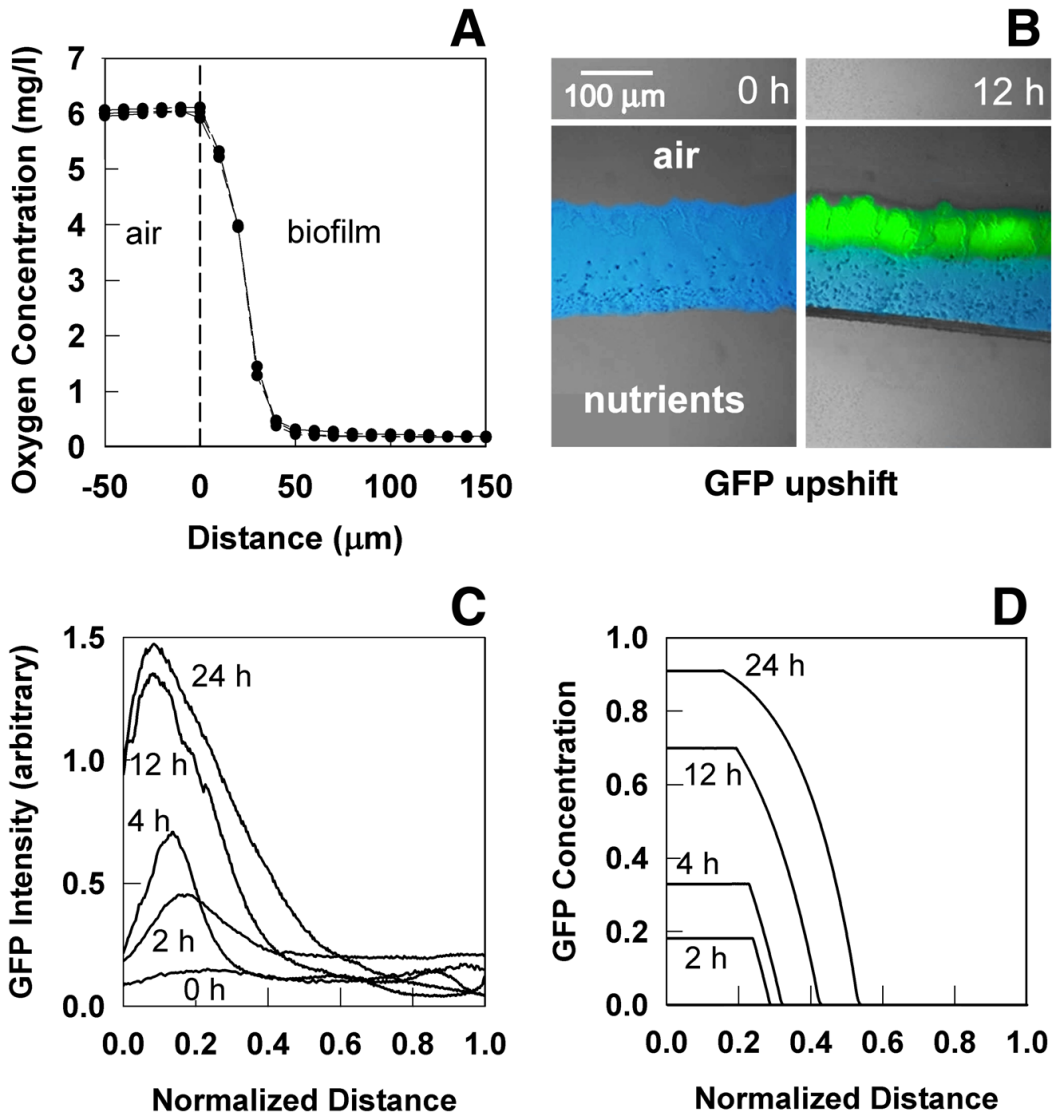
Analysis of GFP expression in *P. aeruginosa* colony biofilms. Biofilms were developed for 48 h on membranes resting on tryptic soy agar plates. The two papers from which the experimental data in this figure were drawn used identical experimental systems [33], [34]. A, oxygen concentration profile in mature colony biofilm. Reprinted with permission from [34]; B, experimental GFP distribution (green) in frozen sections prior to induction (B1) and at 12 h post induction (B2). The transmission image in panel B1 was false colored blue to indicate the extent of biomass more clearly. The polymer membrane supporting the colony biofilm appears as a dark stripe in panel B2; the membrane detached from the biofilm specimen shown in panel B1 and so is absent. Reprinted with permission from [33]; C, Experimentally measured GFP fluorescent intensity in transects within the biofilm at various time points following induction (addition of arabinose). Zero on the x-axis corresponds to the biofilm-air interface; D, simulated GFP fluorescent intensity within the biofilm at various time points following induction. In panels C and D the air interface of the biofilm was on the left.

Inducible fluorescent protein constructs have been used to visualize stratified growth in biofilms [33], [34]. When the inducer is added to a mature biofilm, only those bacteria with the capacity for de novo protein synthesis express the fluorescent protein. In biofilms formed by the aerobe *P. aeruginosa*, this zone corresponds to the oxic region of the biofilm (Figure 2A and 2B). We approximated the experimental oxygen concentration profile by adjusting the reaction rate parameter, then predicted the evolution of a stable induced GFP. The theoretical prediction (Figure 2D) qualitatively captures the time-dependent increase in fluorescent intensity and expansion of the width of the fluorescent band that was experimentally observed (Figure 2C). The shapes of the simulated fluorescent intensity peaks do not match the experimentally measured patterns exactly because the air boundary of the biofilm (corresponding to 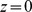 in the figure) is not perfectly flat, the cell density near the air interface may not be uniform, and the assumption of zero-order kinetics is an idealization.

**Figure 3:**
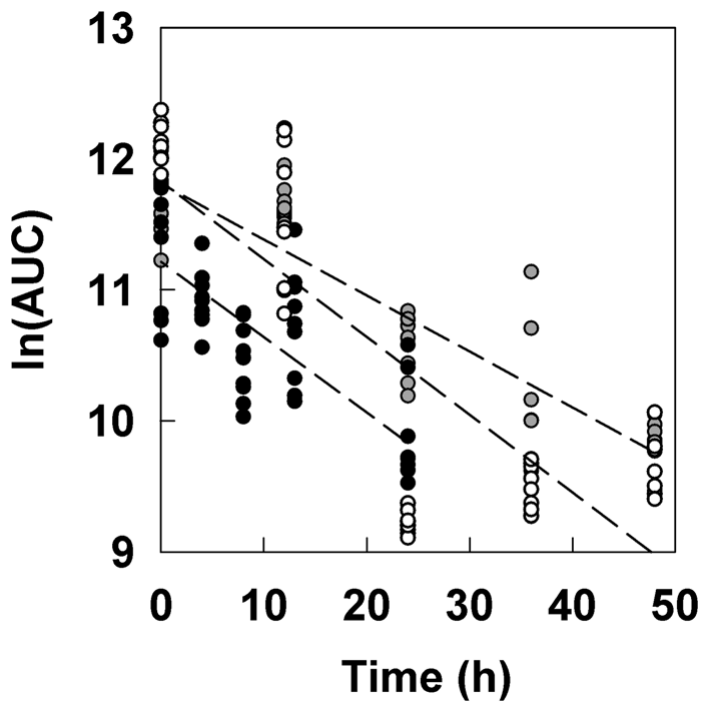
Decay of GFP fluorescence in *P. aeruginosa* colony biofilms. The integrated fluorescence or area under the curve (AUC) was determined. Colony biofilms were grown under GFP-inducing conditions for 48 h then transferred to non-inducing conditions at time zero. Data from three replicate experiments are shown (symbols). Dashed lines are least-squares regressed lines to each of the experimental data sets. The negative slope of each line yields an estimate of the turnover rate coefficient, 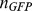.

An experimental protocol in which the biofilm is grown under continuous induction of GFP expression, then transferred to medium lacking the inducer gives access to the turnover rate of the protein. When the synthesis term is set to zero and equation 6 is integrated with respect to the spatial variable 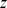 from 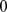 to 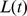, by the Reynolds transport theorem, the result is:
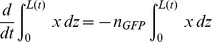



Analysis of experimental data (Figure 3) indicates a mean turnover rate for GFP in this system of 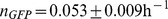. This result shows that even a stable GFP decays in time.

**Figure 4:**
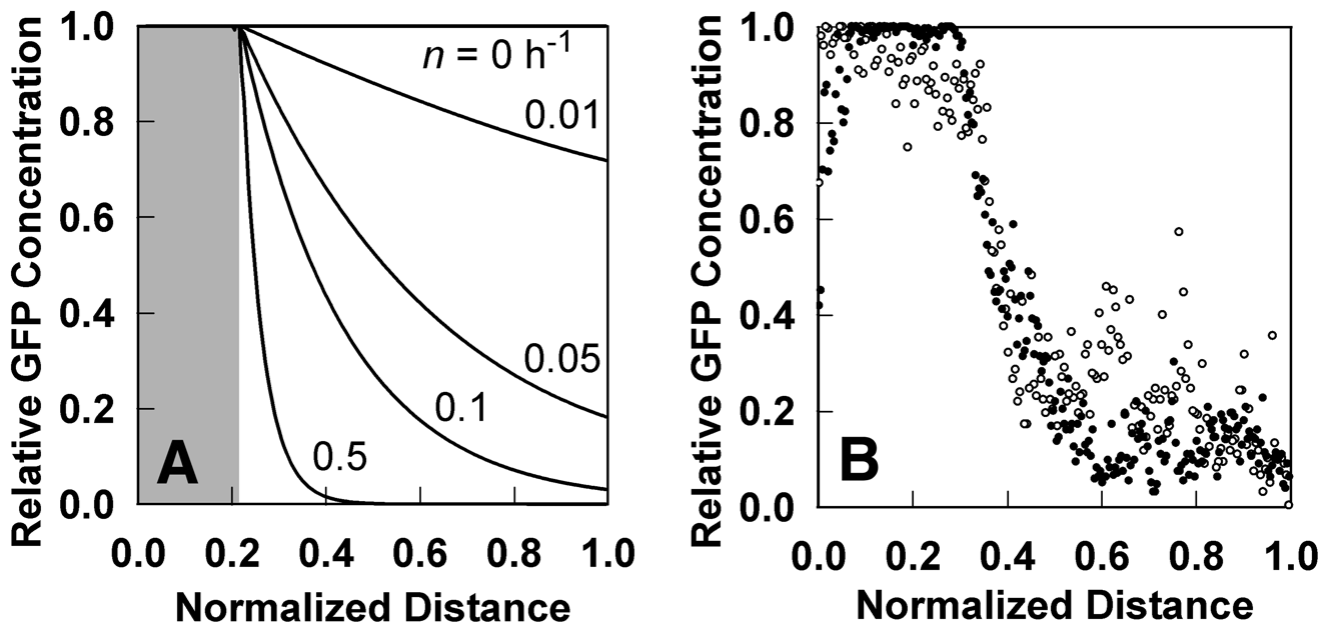
Spatial pattern of GFP fluorescence within a biofilm as a function of GFP stability. A, predicted GFP pattern dependence on the value of the turnover rate coefficient, 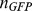. Grey shading indicates the growing region; outside this zone there is no growth because oxygen has been depleted. B, experimentally measured GFP distribution for an unstable GFP in a *P. aeruginosa* colony biofilm [34]. Results from two locations in the biofilm section in Figure 4C of [34] are shown.

The experiment analyzed in Figure 3 made use of a stable GFP. To explore the effect of the stability of the protein we simulated the expression of a protein from a constitutive promoter and varied the turnover rate coefficient, 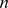 (Figure 4A). The resulting spatial pattern is highly dependent on reporter protein stability. The more stable the protein, the more uniformly it is distributed throughout the biofilm. Indeed, if the fluorescent protein is perfectly stable (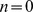) the ability to discern spatial patterns (in this case, to identify the growing region corresponding to the shaded area of Figure 4A) is completely lost. The more unstable the protein, the more accurately it maps the region of active protein synthesis. But even an unstable fluorescent protein reporter overestimates the actual region of gene expression or growth. The delay that results from the finite decay time of the protein means that fluorescent protein reporters can show activity in regions of the biofilm where in fact there is little or none. Intensity profiles for an unstable GFP reporter in a *P. aeruginosa* colony biofilm are shown for comparison (Figure 4B). The data from Figure 4B indicate a turnover rate of approximately 0.2 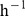.

**Figure 5:**
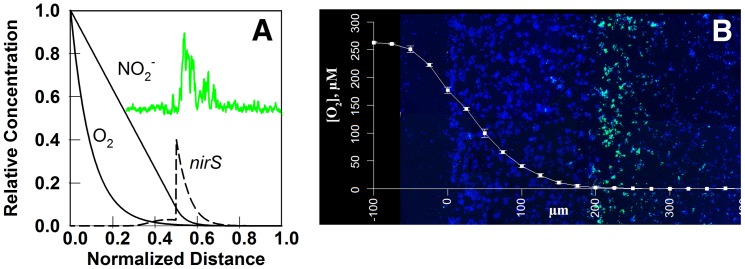
Simulated distribution of *nirS* mRNA in *P. stutzeri* artificial biofilm and comparison to experimental data. A, simulated oxygen, nitrite, and predicted *nirS* mRNA distribution after 48 h. Shown in green is the *nirS* probe signal quantified from the image in panel B. The baseline and height are arbitrary. B, experimentally reported oxygen profile and *nirS* pattern demonstrated by FISH. Reprinted with permission from [36]. Panel B shows a cross section of an artificial biofilm consisting of bacteria immobilized in agarose gel which was incubated in a medium containing oxygen and nitrite. The medium was on the left. Blue color (DAPI) indicates a relatively uniform distribution of biomass with depth while green color is from the *nirS*-specific probe.

Inspired by the recent application of fluorescent in-situ hybridization (FISH) to localize the mRNA for a particular gene [36], we simulated the expression of the *nirS* gene in a *P. stutzeri* biofilm (Figure 5). The simulation correctly captures the expression of the gene in an internal stratum of the biofilm. This region corresponds to that in which the oxygen concentration is very low yet nitrite concentration is sufficient to allow for cell growth. In the oxygen-replete region adjacent to the oxygen source (corresponding to distances between approximately 0 and 200 microns in depth in Fig. 5B), abundant oxygen represses expression of *nirS*. In the region beyond about 300 microns in depth in Fig. 5B, both oxygen and nitrite have been depleted. Although in this region repression by oxygen is alleviated, there is no cellular growth to support gene transcription. Note that the simulated nitrite concentration profile in Fig. 5A constitutes a testable prediction.

**Figure 6:**
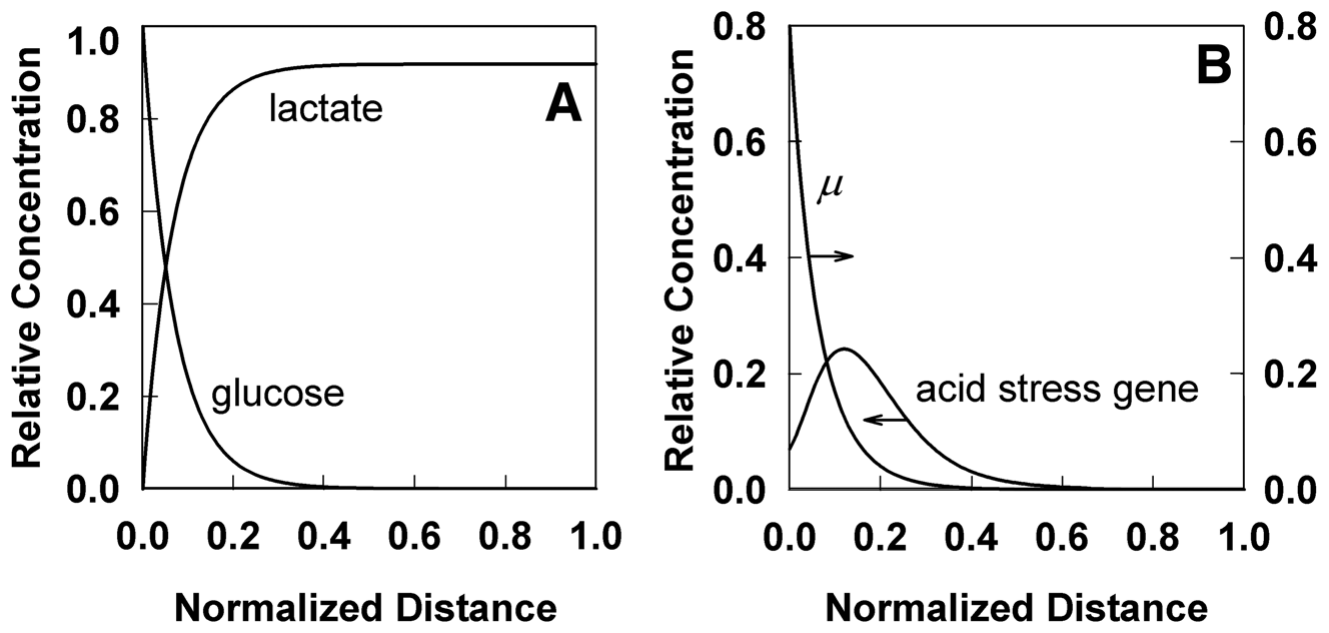
Simulated acid stress response in a *Staphylococcal* biofilm fermenting glucose to lactate. A, glucose and lactate concentrations. B, specific growth rate (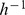) and predicted spatial distribution of an acid stress response gene. Shown are results for the 48 h time point.

The preceding example considered a gene whose expression is repressed by a substrate (oxygen). In the next example, a gene whose expression is induced by a metabolic product is examined. Specifically, we consider the response to acid stress which might occur when a staphylococcal biofilm ferments sugar to an acidic product such as lactic acid [43]. Urease would be an example of such an acid-induced gene [44], [45]. The predicted spatial pattern of expression of such a gene shows a peak with a maximum just beneath the biofilm-bulk fluid interface (Figure 6). Note that urease has been reported to be differentially expressed in biofilms [6], [11], but there are no experimental measurements of its spatial localization. The result in Figure 6B offers a testable prediction.

**Figure 7:**
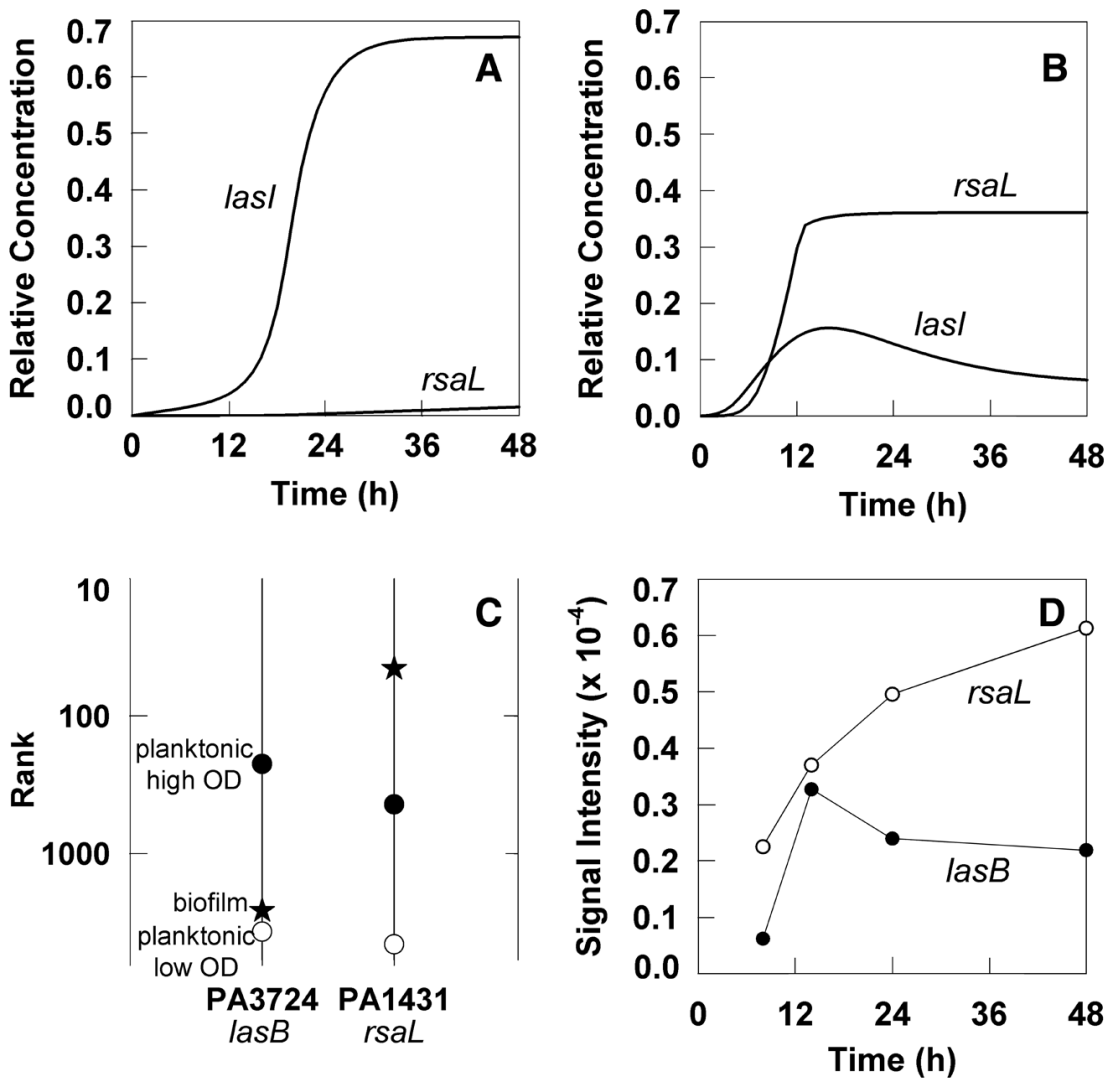
Simulated quorum sensing-regulated expression for *lasI* and *rsaL* genes. The simulations used the same parameter values in batch (A) and biofilm (B) cultures. C, rank of the *lasB* and *rsaL* gene transcripts for a low optical density planktonic culture of *P. aeruginosa* (open circle, [41]), a high optical density planktonic culture (filled circle, [48]), and a mature biofilm (star, [9]). A low numerical value of rank corresponds to high level of expression. D, microarray signal intensity (a direct measure of the level of gene expression) for the *lasB* and *rsaL* transcripts in a *P. aeruginosa* biofilm during development; data from [49]. Data for *lasB* were used rather than *lasI* because they had a larger dynamic range.

Finally, we have analyzed a quorum sensing circuit with features of both positive and negative feedback. When an aerated batch culture is simulated, the system exhibits the classic “density dependent” gene expression. The *lasI* gene rises from a low level of expression in a low density culture to a high level of expression in an older, dense culture (Figure 7A). The expression of *rsaL* remains low throughout. A qualitatively different result is obtained when simulating a biofilm using identical parameter values. Now *rsaL* expression exceeds *lasI* and *lasI* exhibits a maximum, diminishing as the biofilm ages (Figure 7B). Comparison to experimental data suggest that the simulated outcomes are plausible (Figure 7C and 7D). The preliminary result in Figure 7B, in which acyl-homoserine lactone quorum sensing peaks then declines during biofilm maturation constitutes a novel hypothesis. The hypothesis is that quorum sensing-dependent gene expression is repressed during maturation of *P. aeruginosa* biofilms due to increased expression of *rsaL*, which is induced by oxygen limitation and subsequently negatively regulates quorum sensing-controlled genes.

## Discussion

Microorganisms in biofilms differ in growth, metabolism, and gene expression in comparison to planktonic cells. Many of these differences can be attributed to alterations in the local environmental chemistry arising from reaction-diffusion interactions. We have derived a general theory for the quantitative analysis of these spatial and temporal patterns and illustrated its applicability with a variety of case studies. The broad extension of reaction-diffusion modeling to the analysis of individual genes or gene networks is an important advance that dovetails with the expanding toolkit of molecular and genetic experimental techniques.

A common feature of the simulated cases is stratified growth restricting mRNA or protein synthetic activity to regions of the biofilms receiving sufficient nutrients. When this pattern of growth is integrated with a particular environmental chemistry affecting gene or protein expression, unique spatial patterns in the distribution of the products result. In the cases of a gene induced by limitation for a metabolic substrate or accumulation of a metabolic product, the gene is predicted to attain maximal expression at an internal stratum of the biofilm. Such patterns have been observed experimentally [2]–[4].

We also show that the patterns of activity revealed by fluorescent protein constructs overestimate the true region of activity. This is due to advective spreading of biomass and the finite turnover rate of the fluorescent protein. These theoretical predictions can aid in the interpretation of experimental measurements of real-time gene expression using, for example, unstable GFP reporters. The GFP turnover rates we estimate by comparison to experimental patterns in biofilms are of the same order of magnitude of those previously reported [46].

Complex outcomes are possible when considering interacting genes. Already with just two genes and two substrates simulating a simple quorum sensing system, we arrive at non-intuitive outcomes that are qualitatively different from those seen in a planktonic culture at any stage. Modeling will become an important analytical tool to understand the behavior of networks of multiple genes and substrates in biofilms [47].

An implicit assumption in the models presented in this article, and also of prior biofilm models, is that bacterial activities are dictated solely by the local chemical microenvironment. In other words, solving reaction-diffusion problems to determine this microenvironment should be sufficient to describe the biology within the biofilm. Another way to say this is that a cell in a biofilm is no different from a planktonic cell. Both cells simply respond to the chemistry of the environment that is presented to them. This assumption has been sufficient to explain the salient features of many real world phenomena as outlined in the Introduction.

There may be microbial sensing mechanisms beyond those that detect dissolved solutes which contribute to differential gene expression in biofilms. These could include sensing of extracellular polymeric substances or molecules on the surfaces of neighboring cells or sensing of the mechanical environment as by the resistance to movement of a motility appendage. Such mechanisms are not accounted for in the current version of our theory but invite investigation. What changes in biofilm gene expression cannot be explained by accounting for the local concentrations of solutes?

## Conclusion

We conclude that simulation and prediction of spatial and temporal dynamics of gene expression in biofilms is mathematically tractable and a potentially fruitful approach to gain insight into the physiology, metabolism, and gene regulation of microorganisms in biofilms.

## Supporting Information

Figure S1
**Assumed functional dependencies of** 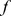 **, the fraction of total mRNA synthetic activity devoted to a particular gene, in three case studies.** A, *nirS* gene expression as a function of oxygen concentration (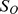). B, acid stress response gene expression as a function of the concentration of the acidic product lactate (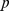). C, expression of the *lasI* autoinducer synthase gene as a function of the autoinducer concentration (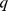). Mathematical statements of these functions are given in the respective case study descriptions in the Materials and Methods.(TIF)

Table S1 **Nomenclature.**
(PDF)

Table S2 **Parameter values for Inducible GFP simulation.**
(PDF)

Table S3 **Parameter values for Denitrification simulation.**
(PDF)

Table S4 **Parameter values for Acid Stress Response simulation.**
(PDF)

Table S5 **Parameter values for Quorum Sensing simulation.**
(PDF)

Text S1 **Change of coordinate and references for parameter values.**
(PDF)
