# Supplementary figures and images for: General Theory for Integrated Analysis of Growth, Gene, and Protein Expression in Biofilms
Source: PLoS One. 2013 Dec 23;8(12):e83626. doi: 10.1371/journal.pone.0083626 (PMC3871705; doi:10.1371/journal.pone.0083626)

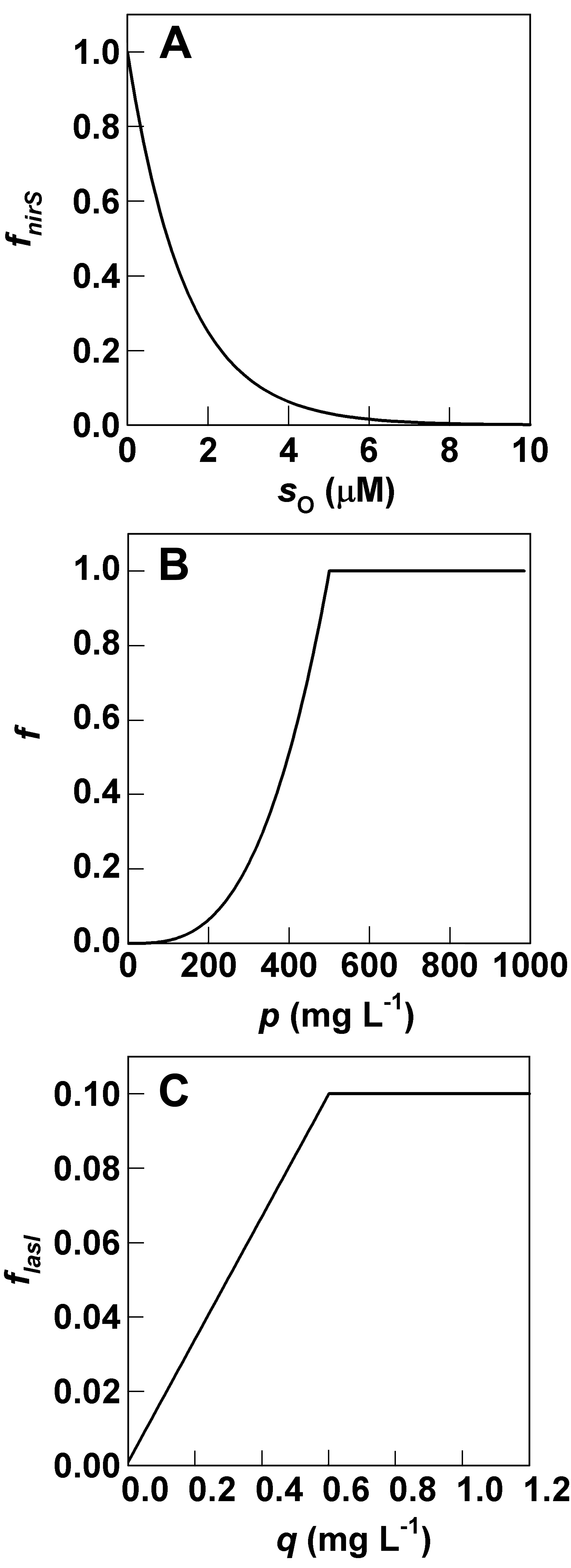

Supplement: Figure S1 — Assumed functional dependencies of , the fraction of total mRNA synthetic activity devoted to a particular gene, in three case studies. A, nirS gene expression as a function of oxygen concentration (). B, acid stress response gene expression as a function of the concentration of the acidic product lactate (). C, expression of the lasI autoinducer synthase gene as a function of the autoinducer concentration (). Mathematical statements of these functions are given in the respective case study descriptions in the Materials and Methods. (TIF) [file pone.0083626.s001.tif]
