# Supplementary material for: General Theory for Integrated Analysis of Growth, Gene, and Protein Expression in Biofilms
Source: PLoS One. 2013 Dec 23;8(12):e83626. doi: 10.1371/journal.pone.0083626 (PMC3871705; doi:10.1371/journal.pone.0083626)
Supplement: Table S1 — Nomenclature. (PDF) [file pone.0083626.s002.pdf]

Symbol	Parameter	Unit
$D,\ D_i$	Metabolite diffusion coefficient in biofilm	$L^2 \cdot T^{-1}$
J	Number of metabolites	
L	Biofilm thickness	L
L_0	Initial biofilm thickness	L
M	Number of genes (mRNA)	
N	Number of proteins	
p	Product concentration	$M \cdot V^{-1}$
P	Overall protein synthesis rate	$M \cdot V^{-1} \cdot T^{-1}$
R	Biofilm cluster radius	L
X	Cell density	$M \cdot V^{-1}$
Y_{xs}	Yield coefficient of biomass on nutrient s	$(\mathrm{M\ X})(\mathrm{M\ s})^{-1}$
Y_{ps}	Yield coefficient of product on nutrient s	$(\mathrm{M\ p})(\mathrm{M\ s})^{-1}$
$f,\ f_i$	Fraction of total mRNA synthesis	Dimensionless
$g,\ g_i$	Fraction of total protein synthesis	Dimensionless
h_0	Characteristic length scale	L
k	Reaction rate coefficient for metabolite	T^{-1} or $M \cdot T^{-1}$
$k_L a$	Oxygen gas-liquid mass transfer coefficient	T^{-1}
m_i	Turnover rate of i-th mRNA	T^{-1}
n_i	Turnover rate of i-th protein	T^{-1}
q	Quorum sensing molecule concentration	$M \cdot V^{-1}$
r	Spatial variable for spherical symmetry case	
$s,\ s_i$	Metabolite concentration	$M \cdot V^{-1}$
s^0	Bulk concentration of nutrient	$M \cdot V^{-1}$
t	Time variable	
t_0	Characteristic time scale	T
v	Biofilm growth velocity	$L \cdot T^{-1}$
$w,\ w_i$	Gene transcript (mRNA) concentration	$M \cdot V^{-1}$
$x,\ x_i$	Protein concentration	$M \cdot V^{-1}$
$\vec{y}$	General spatial variable	
z	Spatial variable in 1D	
α_i	Controlling parameter in quorum sensing	
β, β_i	Metabolite reaction rate	$M \cdot V^{-1} \cdot T^{-1}$
η	Controlling parameter in denitrification	$M^{-1} \cdot V^3$
θ	Overall gene synthesis rate	$M \cdot V^{-1} \cdot T^{-1}$
μ	Growth rate of biofilm	T^{-1}
μ_{max}	Maximum specific growth rate (0-order kinetic)	T^{-1}
μ_I	Growth rate coefficient (1st-order kinetic)	$T^{-1} \cdot M^{-1} \cdot V$
ρ_R	mRNA density	$M \cdot V^{-1}$
ρ_P	Protein density	$M \cdot V^{-1}$
σ	Biofilm detachment coefficient	$L^{-1} \cdot T^{-1}$

Table S1: Nomenclature. Notations for units: M = [mass], L = [length], T = [time], V = [volume].
