# Supplementary material for: General Theory for Integrated Analysis of Growth, Gene, and Protein Expression in Biofilms
Source: PLoS One. 2013 Dec 23;8(12):e83626. doi: 10.1371/journal.pone.0083626 (PMC3871705; doi:10.1371/journal.pone.0083626)
Supplement: Table S2 — Parameter values for Inducible GFP simulation. (PDF) [file pone.0083626.s003.pdf]

Symbol	Parameter	Value	Ref.
D	Oxygen diffusion coefficient	$1.53 \times 10^{-9}\ \mathrm{m}^2 \cdot \mathrm{s}^{-1}$	[1]
L_0	Initial biofilm thickness	$139\ \mu\mathrm{m}$	[2]
g	Fraction of total protein synthesis	0 or 1	*
h_0	Characteristic length scale	$100\ \mu\mathrm{m}$	*
k	Oxygen reaction rate (zero order)	$15\ \mathrm{mg} \cdot \mathrm{l}^{-1} \cdot \mathrm{s}^{-1}$	[1]
n_{GFP}	Turnover rate of GFP	$0 \sim 0.5\ \mathrm{h}^{-1}$	*
s^0	Bulk concentration of oxygen	$6\ \mathrm{mg} \cdot \mathrm{l}^{-1}$	[2]
t_0	Characteristic time scale	$3600\ \mathrm{s}$	*
μ_{max}	Maximum specific growth rate of biofilm	$0.1\ \mathrm{h}^{-1}$	†
ρ_P	Protein density	$6\ \mathrm{g} \cdot \mathrm{l}^{-1}$	[1]

Table S 2: Parameter values for Inducible GFP simulation. *: assumed, †: unpublished estimate, other references are listed in **Text S1**.
