# Supplementary material for: General Theory for Integrated Analysis of Growth, Gene, and Protein Expression in Biofilms
Source: PLoS One. 2013 Dec 23;8(12):e83626. doi: 10.1371/journal.pone.0083626 (PMC3871705; doi:10.1371/journal.pone.0083626)
Supplement: Table S3 — Parameter values for Denitrification simulation. (PDF) [file pone.0083626.s004.pdf]

Symbol	Parameter	Value	Ref.
D_O	Oxygen diffusion coefficient	$1.53 \times 10^{-9}\ \mathrm{m}^2 \cdot \mathrm{s}^{-1}$	[1]
D_N	Nitrite diffusion coefficient	$1.32 \times 10^{-9}\ \mathrm{m}^2 \cdot \mathrm{s}^{-1}$	[4]
L	Biofilm thickness (constant)	$400\ \mu\mathrm{m}$	[3]
h_0	Characteristic length scale	$100\ \mu\mathrm{m}$	*
k_O	Oxygen reaction rate (first order)	$1.02\ \mathrm{s}^{-1}$	*
k_N	Nitrite reaction rate (first order)	$4\ \mathrm{s}^{-1}$	*
s_O^0	Bulk concentration of oxygen	$175\ \mu\mathrm{mol} \cdot \mathrm{l}^{-1}$	[3]
s_N^0	Bulk concentration of nutrient	$5\ \mathrm{mmol} \cdot \mathrm{l}^{-1}$	[3]
t_0	Characteristic time scale	$3600\ \mathrm{s}$	*
η	Parameter controlling oxygen interactions	$\log 2\ \mu\mathrm{mol}^{-1} \cdot \mathrm{l}$	*
μ_O	Specific growth rate consuming oxygen	$0.1/175\ \mathrm{h}^{-1} \cdot (\mu\mathrm{mol\ oxygen})^{-1} \cdot \mathrm{l}$	*
μ_N	Specific growth rate consuming nitrite	$0.1/5\ \mathrm{h}^{-1} \cdot (\mathrm{mmol\ nitrite})^{-1} \cdot \mathrm{l}$	*
ρ_R	mRNA density	$6\ \mathrm{g} \cdot \mathrm{l}^{-1}$	†

Table S 3: Parameter values for Denitrification simulation. *: assumed, †: unpublished estimate, other references are listed in **Text S1**.
