# Supplementary material for: General Theory for Integrated Analysis of Growth, Gene, and Protein Expression in Biofilms
Source: PLoS One. 2013 Dec 23;8(12):e83626. doi: 10.1371/journal.pone.0083626 (PMC3871705; doi:10.1371/journal.pone.0083626)
Supplement: Table S4 — Parameter values for Acid Stress Response simulation. (PDF) [file pone.0083626.s005.pdf]

Symbol	Parameter	Value	Ref.
D_s	Glucose diffusion coefficient	$2.24 \times 10^{-10}\,\mathrm{m}^2 \cdot \mathrm{s}^{-1}$	[1]
D_p	Lactate diffusion coefficient	$3.51 \times 10^{-10}\,\mathrm{m}^2 \cdot \mathrm{s}^{-1}$	[1]
L_0	Initial biofilm thickness	$80\,\mu\mathrm{m}$	*
R_p	Penetration depth of glucose	$100\,\mu\mathrm{m}$	*
X	Cell density	$5\,\mathrm{g} \cdot \mathrm{l}^{-1}$	*
Y_{xs}	Yield coefficient of biomass on glucose	$0.5\,(\mathrm{g\ X})(\mathrm{g\ s})^{-1}$	[5]
Y_{ps}	Yield coefficient of lactate on glucose	$0.9\,(\mathrm{g\ p})(\mathrm{g\ s})^{-1}$	[5]
h_0	Characteristic length scale	$100\,\mu\mathrm{m}$	*
m	Turnover rate of gene	$0.1\,\mathrm{h}^{-1}$	*
s^0	Bulk concentration of glucose	$800\,\mathrm{mg} \cdot \mathrm{l}^{-1}$	†
t_0	Characteristic time scale	$3600\,\mathrm{s}$	*
μ_I	Specific growth rate coefficient	$0.001\,\mathrm{h}^{-1} \cdot \mathrm{mg}^{-1} \cdot \mathrm{l}$	*
ρ_R	mRNA density	$6\,\mathrm{g} \cdot \mathrm{l}^{-1}$	*
σ	Biofilm detachment coefficient	$0.345\,\mathrm{m}^{-1} \cdot \mathrm{s}^{-1}$	*

Table S4: Parameter values for Acid Stress Response simulation. *: assumed, †: blood plasma concentration, other references are listed in **Text S1**.
