# Supplementary material for: General Theory for Integrated Analysis of Growth, Gene, and Protein Expression in Biofilms
Source: PLoS One. 2013 Dec 23;8(12):e83626. doi: 10.1371/journal.pone.0083626 (PMC3871705; doi:10.1371/journal.pone.0083626)
Supplement: Table S5 — Parameter values for Quorum Sensing simulation. (PDF) [file pone.0083626.s006.pdf]

Symbol	Parameter	Value	Ref.
D_s	Oxygen diffusion coefficient	$1.53 \times 10^{-9}\,\mathrm{m}^2 \cdot \mathrm{s}^{-1}$	[1]
D_q	QS molecule diffusion coefficient	$1.2 \times 10^{-10}\,\mathrm{m}^2 \cdot \mathrm{s}^{-1}$	[1]
L_0	Initial biofilm thickness	$10\,\mu\mathrm{m}$	*
Y_{xs}	Yield coefficient of biomass on oxygen in batch	$0.5\,(\mathrm{g\ X})(\mathrm{g\ s})^{-1}$	[6]
h_0	Characteristic length scale	$100\,\mu\mathrm{m}$	*
$k_L a$	Oxygen gas-liquid mass transfer coefficient	$0.01\,\mathrm{s}^{-1}$	*
q_0	Critical q value in defining f_{lasI}	$0.6\,\mathrm{mg} \cdot \mathrm{l}^{-1}$	*
s^0	Bulk concentration of oxygen	$6\,\mathrm{mg} \cdot \mathrm{l}^{-1}$	‡
t_0	Characteristic time scale	$3600\,\mathrm{s}$	*
α_1	Production of q due to *lasI*	$2 \times 10^4\,(\mathrm{g}\ q) \cdot (\mathrm{g}\ lasI)^{-1}$	*
α_2	Suppression of *lasI* by *rsaL*	$100\,(\mathrm{g}\ rsaL)^{-1} \cdot \mathrm{l}$	*
α_3	Suppression of *rasL* by oxygen	$1\,(\mathrm{g\ oxygen})^{-1} \cdot \mathrm{l}$	*
μ_{max}	Maximum specific growth rate	$0.1\,\mathrm{h}^{-1}$	†
ρ_R	mRNA density	$6\,\mathrm{g} \cdot \mathrm{l}^{-1}$	*

Table S5: Parameter values for Quorum Sensing simulation. *: assumed, †: unpublished estimate, ‡: air saturation, other references are listed in **Text S1**.
