# Supplementary material for: General Theory for Integrated Analysis of Growth, Gene, and Protein Expression in Biofilms
Source: PLoS One. 2013 Dec 23;8(12):e83626. doi: 10.1371/journal.pone.0083626 (PMC3871705; doi:10.1371/journal.pone.0083626)
Supplement: Text S1 — Change of coordinate and references for parameter values. (PDF) [file pone.0083626.s007.pdf]

Change of coordinate

Equations (5) - (8) are to be solved on a domain $z \in [0, L]$ with a moving upper boundary at $z = L(t)$. For computational convenience, we introduce a new spatial coordinate $\zeta = z/L(t)$ in order to maintain a fixed domain $\zeta \in [0, 1]$. The change of coordinate implies the following change in partial derivatives:

$$\frac{\partial}{\partial z} \to \frac{1}{L}\frac{\partial}{\partial \zeta}, \qquad \frac{\partial}{\partial t} \to -\zeta\frac{\dot{L}}{L}\frac{\partial}{\partial \zeta} + \frac{\partial}{\partial t}$$

We also introduce the scaled velocity $u(\zeta) = v(z)/L$. In the new coordinates, equations (5) - (8) become

$$\begin{aligned}
\frac{D_s}{L^2}\frac{d^2 s}{d\zeta^2} &= \beta(s) \\
\frac{\partial x}{\partial t} &= g \cdot P - \frac{\partial(xu)}{\partial \zeta} + \zeta\frac{\dot{L}}{L}\frac{\partial x}{\partial \zeta} - n_{GFP} \cdot x \\
\frac{du}{d\zeta} &= \mu(s) \\
\frac{dL}{dt} &= Lu(\zeta, t)
\end{aligned}$$

The same change of coordinate is applied in solving equations (12) - (16) and (17) - (22).

References

[1] Stewart PS (2003) Diffusion in biofilms. J Bacteriol 185: 1485-1491

[2] Werner E, Roe F, Bugnicourt A, Franklin MJ, Heydorn A, Molin S, Pitts B, Stewart PS (2004) Stratified growth in *Pseudomonas aeruginosa* biofilms. Appl Environ Microbiol 70: 6188-6196

[3] Kofoed MVW, Nielsen DA, Revsbech NP, Schramm A (2012) Fluorescence in situ hybridization (FISH) detection of nitrite reductase transcripts (nirS mRNA) in *Pseudomonas stutzeri* biofilms relative to a microscale oxygen gradient. Syst Appl Microbiol 35: 513-517

[4] Stewart PS (1998) A review of experimental measurements of effective diffusive permeabilities and effective diffusion coefficients in biofilms. Biotechnol Bioeng 59: 261-272

[5] Sivakanesan R, Dawes EA (2000) Anaerobic glucose and serine metabolism in *Staphylococcus epidermidis*. J Gen Microbiol 118: 143-157

[6] Bailey JE, Ollis DF (1986) Biochemical engineering fundamentals. McGraw-Hill, New York.
